# Pharmacological Mobilization of Endogenous Bone Marrow Stem Cells Promotes Liver Regeneration after Extensive Liver Resection in Rats

**DOI:** 10.1038/s41598-018-21961-2

**Published:** 2018-02-26

**Authors:** Rujun Zhai, Yongchun Wang, Le Qi, George Melville Williams, Bin Gao, Guang Song, James F. Burdick, Zhaoli Sun

**Affiliations:** 1 Department of Hepatobiliary Surgery, Tianjin Medical University Cancer Institute & Hospital and Tianjin Medical University Graduate School, Tianjin, P.R. China; 2 Department of Surgery, Johns Hopkins University School of Medicine, Baltimore, MD USA; 3 Laboratory of Liver Disease, NIAAA/NIH, Rockville, MD USA; 4 Department of Pharmacology and Molecular Sciences, Johns Hopkins University School of Medicine, Baltimore, MD USA

## Abstract

Rapid regeneration of the remnant liver is critical for preventing liver failure and promoting recovery after extensive liver resection. Numerous studies have demonstrated the involvement of bone marrow-derived stem cells in liver regeneration and the potential benefits of bone marrow stem cell therapy. To avoid the preparation of stem cells, we proposed in this study to mobilize endogenous bone marrow stem cells pharmacologically with a combination of AMD3100 (A), an antagonist of CXCR4 and low-dose FK506 (F). Here we show that AF combination therapy significantly increased lineage negative (Lin-) CD34+ and Lin-CD133+ stem cells in peripheral blood and enhanced recruitment of CD133+ cells into the remnant liver in a rat model of 85% partial hepatectomy. Recruiting CD133+ stem cells in the remnant liver was associated with increased proliferation of hepatic oval cells and paralleled the increased SDF-1, CXCR4 and HGF expression. Importantly, AF combination therapy increased the number of Ki67 positive hepatocytes and BrdU incorporation in the remnant liver and improved serum levels of albumin. Our results demonstrate that pharmacological mobilization of endogenous bone marrow stem cells with AF combination therapy can enhance endogenous stem cell mobilization to promote liver regeneration and improve liver function after extensive hepatectomy.

## Introduction

Liver failure is a severe complication of extensive liver resection especially in patients with active hepatitis, cirrhosis and limited residual liver tissue. The incidence of liver failure after hepatectomy is about 0.70–33.83%^1–5^ and failure is related to inadequate residual liver tissue and functional capacity^6–8^. Rapid regeneration of the remnant liver is critical for preventing liver failure and promoting recovery after liver resection. However, currently no approved therapy is available for accelerating liver regeneration.

Liver regeneration after partial hepatectomy depends on the proliferation of hepatocytes. But in addition, numerous studies have demonstrated the additional involvement of extra-hepatic stem/progenitor cells in liver regeneration^9,10^. Hematopoietic stem cells (HSCs) and mesenchymal stem cells (MSCs) of bone marrow (BM) origin can be induced to differentiate into liver cells *in vitro* and differentiation of BM HSC or MSC into cells of hepatic lineages may also occur *in vivo* in physiological conditions and after liver injury^11–13^. Direct evidence that BM cells participate in liver regeneration after partial hepatectomy has been reported in mice with Green Flourescent Protein (GFP)-BM transplantation^14^ in which a majority of GFP BM cells was committed to form liver sinusoidal endothelial cells (LSECs), an important driver of liver regeneration^15,16^. Further, recruitment of BM progenitors of LSECs to the hepatic sinusoid after partial hepatectomy is required for normal liver regeneration^17^. These findings led to studies using BM-derived HSCs or MSCs. HSCs and MSCs were shown to undergo hepatogenic differentiation and to populate liver after intravenous transplantation in rat, mouse and pig models of liver injury^18–20^. Early results of human trials demonstrated the temporary improvement of MELD score after reinfusion of CD133+ BM cells in patients with end stage liver disease^21,22^ or with liver insufficiency^23^. However, because the preparation of autogenous stem cells has been time consuming and the questions about effective factors for quality and quantity of BM-derived stem/progenitor cells remain unsolved, this approach has limited practical application in the treatment of liver failure. For this reason, the pharmacological amplification of endogenous stem cells is attractive as it provides a simple, rapid means of presenting stem cells to an injured liver.

We discovered a new stem cell mobilizing therapy serendipitously using a combination of two drugs (AMD3100 = A FK506 = F) in animals that prevents organ transplantation rejection^24–26^ and promotes skin wound healing^27^. AMD3100, is a CXCR4 antagonist, originally an anti-HIV medicine but found useful chiefly in the mobilization of CD34 and other stem cells from bone marrow. FK506 is an immunosuppressive drug widely used in solid organ transplantation to overcome organ rejection. We have found a potent, synergistic activity of AMD3100 and low-dose FK506 (one tenth of the dosage used to prevent rejection) in the mobilization and recruitment of BM-derived CD133+ stem cells. With just one week of treatment, the combination of the two drugs (AMD3100 = A FK506 = F, AF) enabled long-term small liver allograft survival and freedom from immunosuppression in an otherwise strongly rejecting rat strain combination^24^. Further, one week of AF combination treatment plus repeat dosing at 1, 2 and 3 months resulted in immunosuppressive drug-free long term kidney allograft survival in rats^25^ and in maximally immunologically mismatched swine^26^. This tolerance was associated with allograft chimerism (host repopulation of the graft) and local down regulation of the immune response in the graft^28–31^. Further, AF treatment also accelerated skin wound healing and promoted hair follicle formation through recruitment of BM-derived stem cells into wound sites^27^. Lineage tracing demonstrated the critical role of CD133 stem cells in enhanced capillary and hair follicle neogenesis, contributing to more rapid and perfect healing.

Here we test the impact on liver regeneration of this stem cell mobilizing therapy using AF combination in a rat model of 85% partial hepatectomy. We found that AF combination therapy was able to promote regeneration and improve functional recovery after extensive liver resection. These results will assist in the development of a therapeutic strategy for liver failure.

## Results

### Pharmacological mobilization of bone marrow stem cells with AF combination therapy improves liver function in rats after extensive liver resection

**Figure 1:**
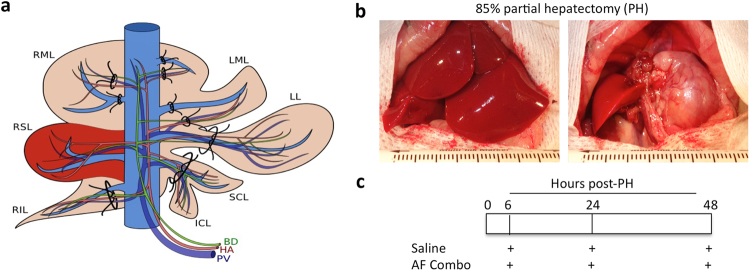
A rat model of 85% partial hepatectomy (PH) and AF treatment protocol. (**a**) Schematic description of vessel-oriented technique for ligating vascular structures within the liver parenchyma separately. Only the right superior lobe (RSL) was retained. (**b**) Intraoperative pictures of the liver before and after resection of defined liver lobes in the 85% PH model. (**c**) Animals were given AF combination or saline subcutaneously at 6, 24 and 48 hours after 85% PH.

**Figure 2:**
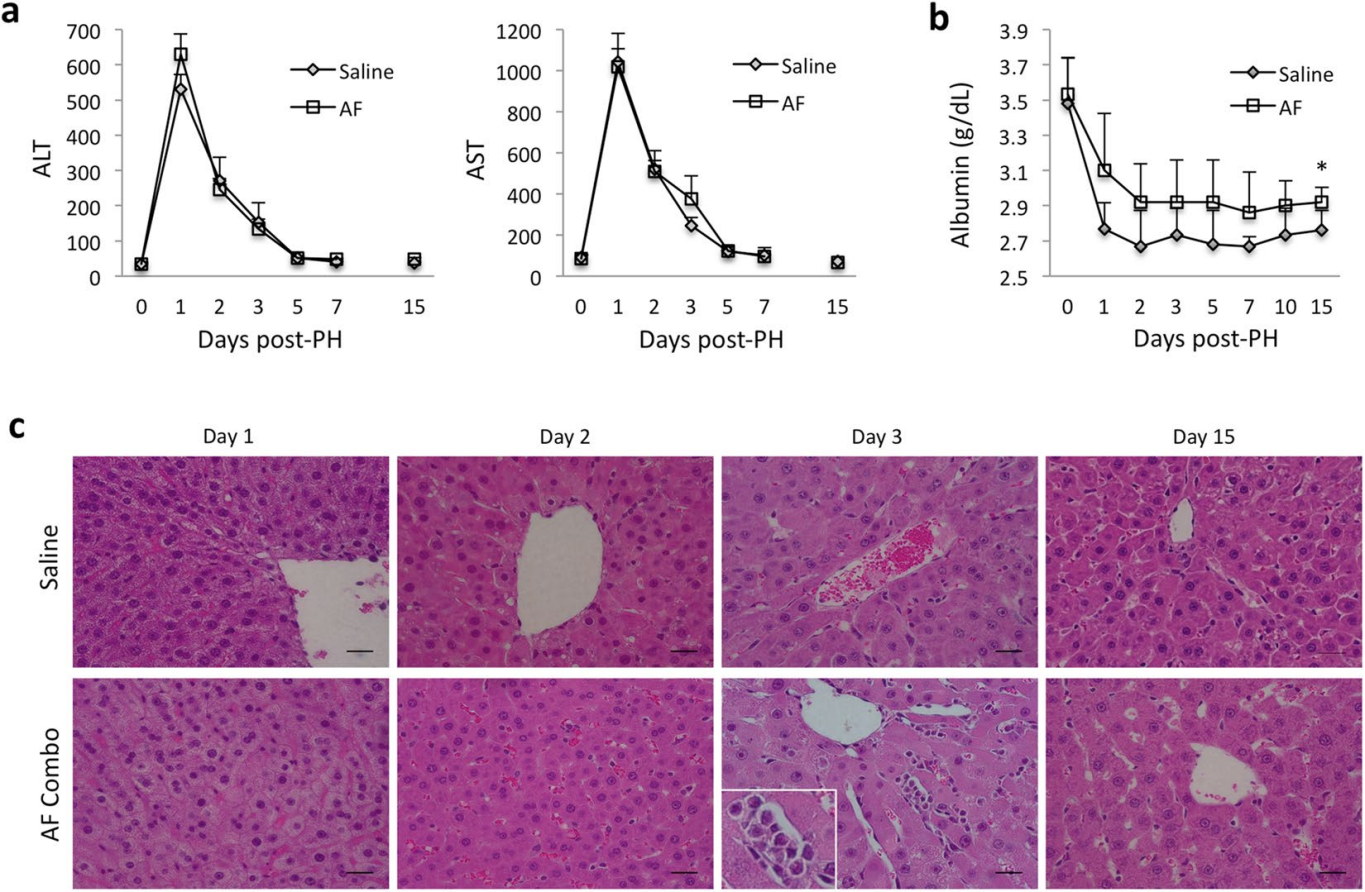
Pharmacological mobilization of bone marrow stem cells with AF combination therapy improves liver function in rats after 85% PH. (**a**) Serum levels of AST and ALT were significantly increased, reaching peak levels at 24 hours and returning to normal levels at 5 days in both groups of animals after 85% PH, suggesting that the liver damage was similar in control and treatment groups. (**b**) Serum albumin levels were decreased at 24 hours and remained at lower levels 15 days after 85% PH. Animals treated with AF combination exhibited higher levels of serum albumin compared to saline treated animals at each time point after 85% PH. Data represent mean ± SE of n = 5 or 6 animals per group. *p < 0.05. (**c**) Liver injury was evaluated in sections stained with hematoxylin-eosin: original magnification, 400x. Scale bar: 50 μm. Day 3, clusters of small mononuclear cells (stem cell like cells) appeared in hepatic sinusoids around central vein areas in tissue sections from animals treated with AF combination.

85% partial hepatectomy was performed in rats by ligating vascular structures within the liver parenchyma separately (Fig. 1a) and only the right superior lobe (RSL) was retained (Fig. 1b). Animals were given AF combination or saline subcutaneously at 6, 24 and 48 hours after 85% PH (Fig. 1c). All animals survived 2 weeks after 85% partial hepatectomy. Serum levels of ALT and AST were significantly increased, reaching peak levels at 24 hour and returning to normal levels at 7 days after 85% PH in both groups (Fig. 2a). Although serum levels of albumin were decreased in both groups after 85% PH, animals treated with the AF combination exhibited higher levels of albumin at each time-point and remained higher at day 15 after 85% PH (Fig. 2b). Histological studies demonstrated liver injury and hepatic steatosis at 1 and 2 days post PH in both groups. Interestingly, clusters of small mononuclear cells (stem cell like cells) appeared in hepatic sinusoids around central vein areas in tissue sections from animals treated with AF combination at 3 days post 85% PH (Fig. 2c). At 15 days post PH, hepatocytic degeneration and hepatocytes with pyknotic nuclei were identified in tissue sections from control animals. In contrast, animals treated with AF combination displayed normal liver histology (Fig. 2c).

### Pharmacological mobilization of bone marrow stem cells with AF combination therapy promotes liver regeneration after extensive liver resection

**Figure 3:**
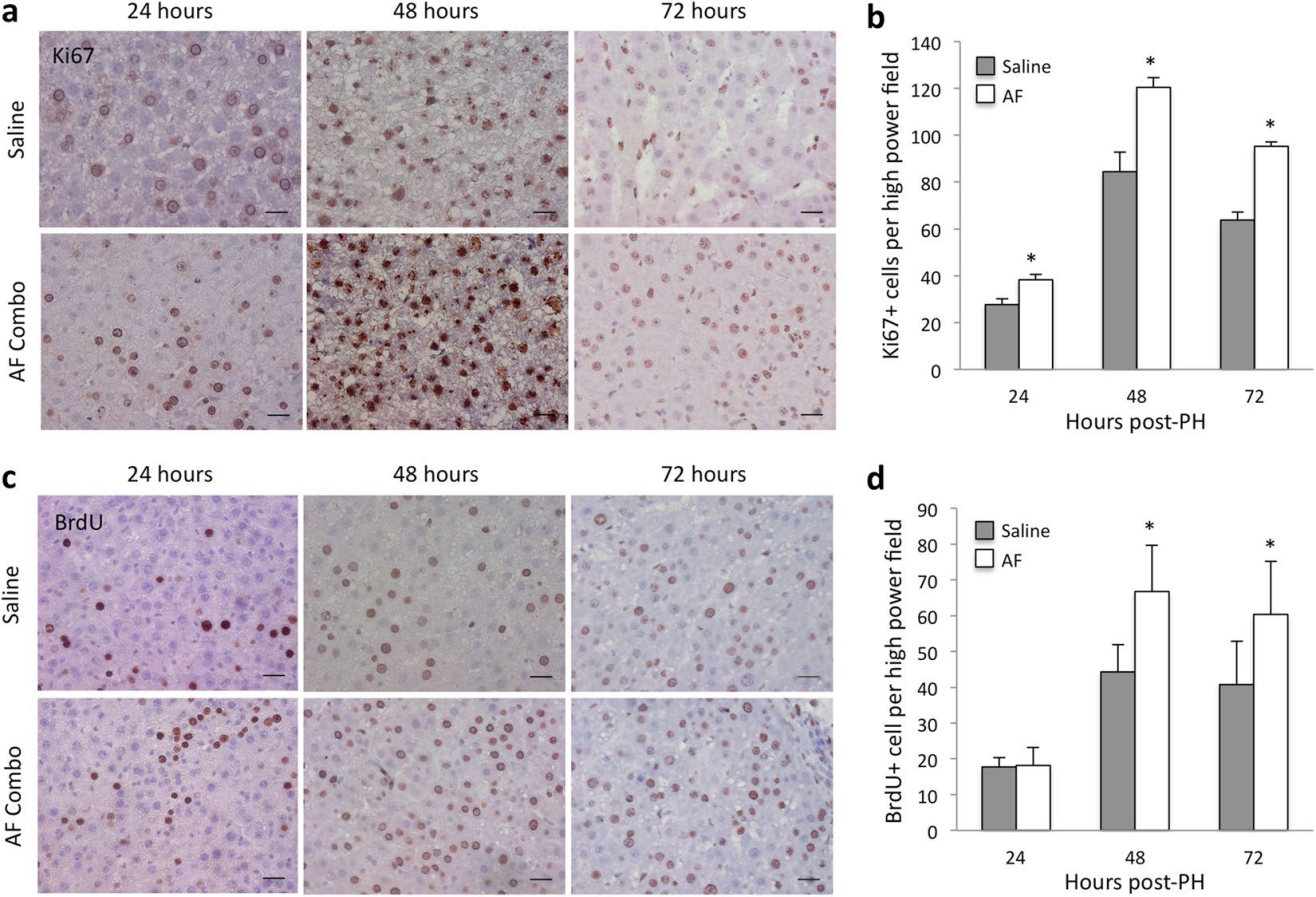
Histologic analysis of Ki67 and BrdU expression in liver sections from animals with or without AF combination treatment after 85% PH. (**a**) Immunohistochemical staining for Ki67. Ki67 positive cells show a brown nuclear pattern. Scale bar: 50 μm. (**b**) Quantitative analysis of Ki67 positive cells per high power field. Data represents mean ± SE of n = 3 or 4 animals per group at each time point. *p < 0.05. (**c**) BrdU staining. BrdU positive cells show brown nuclear stain. Scale bar: 50 μm. (**d**) Quantitative analysis of BrdU positive cells per high power field. Data represents mean ± SE of n = 3 or 4 animals per group at each time point. *p < 0.05.

Immunohistochemistry staining for Ki67 and BrdU incorporation assays were used to evaluate liver regeneration after 85% PH. Ki67 positive cells were significantly increased and reached peak levels in both groups at 48 hours after 85% PH (Fig. 3a). However, the number of Ki67 positive cells was significantly higher in tissue sections from animals treated with the AF combination at 24, 48 and 72 hours after 85% PH (Fig. 3b). Similarly, BrdU incorporation was also increased and reached peak levels at 48 hours after 85% PH (Fig. 3c). Liver tissue sections from animals with AF treatment exhibited significantly higher numbers of BrdU positive hepatocytes at 48 and 72 hours after 85% PH (Fig. 3d).

A replication study by another fellow using an additional twenty animals confirmed that pharmacological mobilization of bone marrow stem cells with AF combination therapy not only promotes liver regeneration but also improves liver function in rats after extensive liver resection (Supplemental Fig. 1).

### Recruitment of mobilized stem cells into the remnant liver following AF combination therapy

**Figure 4:**
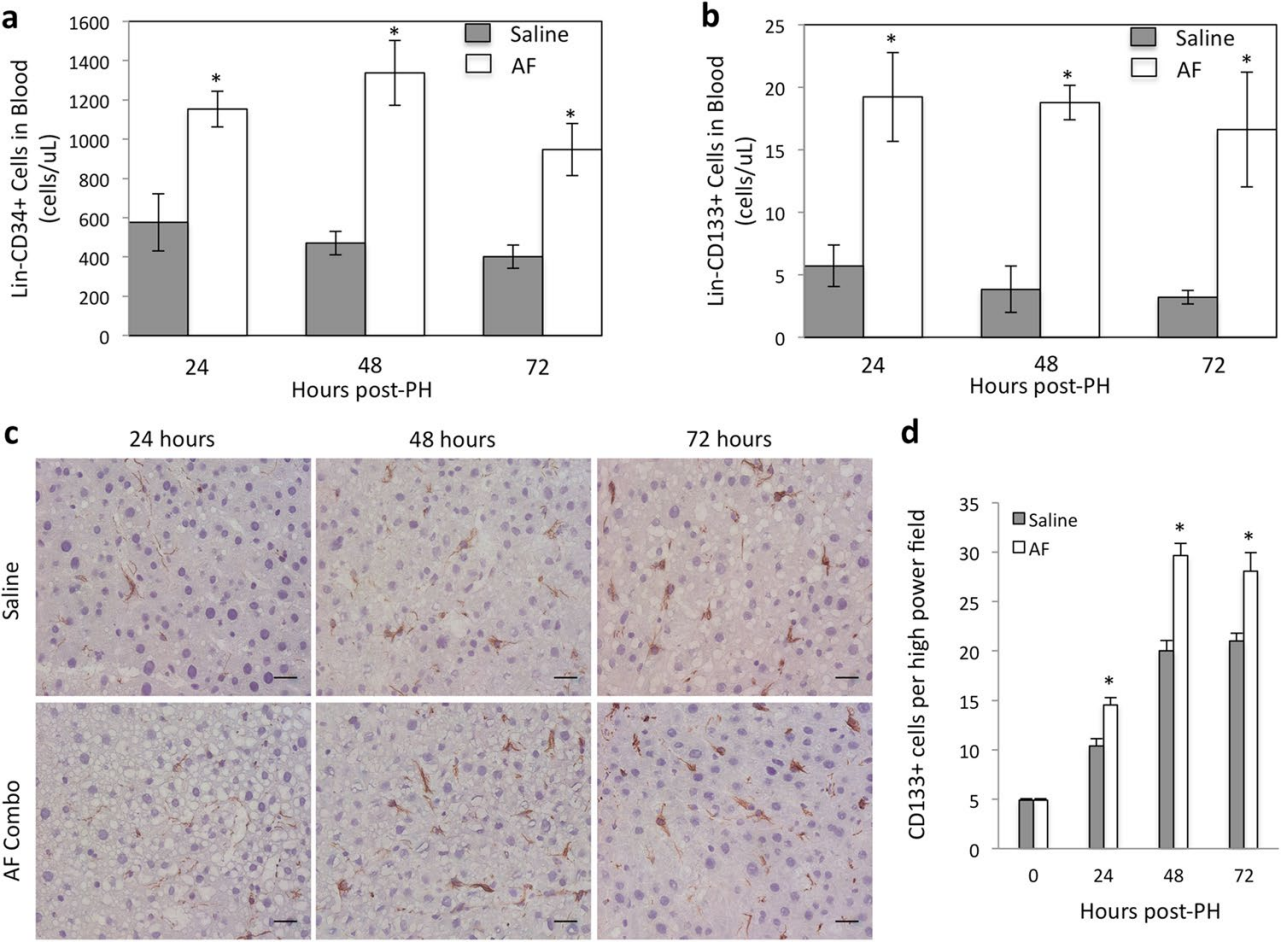
Quantitative analysis of stem cells in peripheral blood and remnant livers after 85% PH. (**a**) and (**b**) Quantitative analysis of Lineage negative (Lin-) CD34+ and CD133+ cells in blood by flow cytometry at 24, 48 and 72 hours after 85% PH. The percentage of Lin-CD34+ and Lin-CD133+ cells was significantly greater in peripheral blood in the animals receiving AF combination treatment. Quantitative data are represented as group means (bars) ± SE (n = 3). *P < 0.01. (**c**) Immunohistochemical staining for CD133. CD133 positive cells are brown. Scale bar: 50 μm. (**d**) Quantitative analysis of CD133+ cells in tissue sections from the remnant liver. The number of CD133+ cells per high power field was significantly higher in the animals receiving AF combination treatment at each time point. Data represents mean ± SE of n = 3 or 4 animals per group at each time point. *p < 0.05.

To determine if AF combination therapy mobilizes bone marrow stem cells in rats after 85% PH, lineage negative (Lin-) CD34+ and Lin-CD133+ stem cells in peripheral blood were analyzed by using flow cytometry and the absolute numbers of Lin-CD34+ or Lin-CD133+ cells in circulating blood were calculated as a percentage of positive cells x WBC counts. Lin-CD34+ cells and Lin-CD133+ cells were increased in the peripheral blood of animals with 85% PH compared to animals without PH (data not shown) supporting the notion that liver injury mobilizes bone marrow stem cells^17,32^. Beyond that, animals treated with AF combination demonstrated significantly increased circulating Lin-CD34+ and Lin-CD133+ stem cells compared with 85% hepatectomized saline controls (Fig. 4a,b).

To determine whether mobilized stem cells are recruited into the remnant liver immunohistochemistry staining for CD133 was performed. Figure 4c shows that CD133+ cells were recognized at 24, 48 and 72 hours after 85% PH in the remnant livers and the number of positive cells increased with time. However, the number of positive cells was significantly higher in tissue sections from animals with AF treatment at each time point after 85% PH (Fig. 4d). These results indicate that AF combination therapy not only mobilized stem cells in the circulation but also increased the recruitment of these stem cells into the remnant liver.

### Recruiting CD133+ stem cells is associated with increased proliferation of hepatic oval cells in animals receiving AF combination therapy after 85% PH

**Figure 5:**
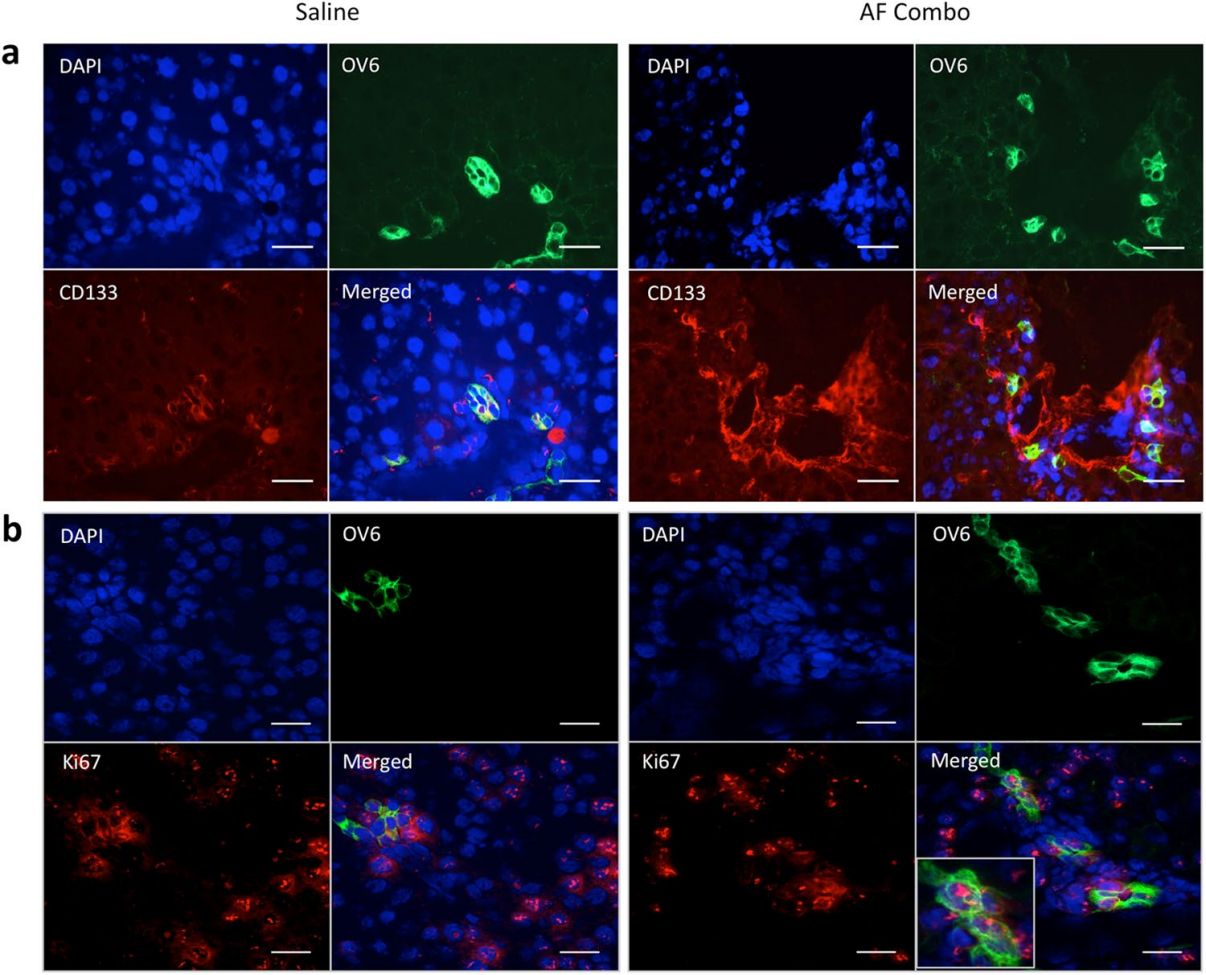
Recruited CD133+ stem cells associated with increased proliferation of hepatic oval cells in animals receiving AF combination at 3 days after 85% PH. (**a**) Double staining for CD133 and OV6, a marker of hepatic oval cells, was performed by immunofluorescence stains using frozen sections. OV6 was stained by anti-OV6 antibody and FITC-conjugated donkey anti mouse IgG (green color), whereas CD133 was stained by anti-CD133 antibody and Cy3-conjugated donkey anti rabbit IgG (red color). CD133 positive cells appeared in the area immediately around OV6 positive cells and both CD133 and OV6 positive cells were increased in animals receiving AF combination therapy. (**b**) Immunofluorescence double staining for OV6 and Ki67. Ki67, a marker of cell proliferation, was stained by anti-Ki67 antibody and Cy3-conjugated donkey anti rabbit IgG (red color). The number of double positive cells in animals receiving AF combination therapy was significantly increased. Cell nuclei were stained blue with DAPI. Original magnification: 400x. Scale bar: 50 μm.

To determine whether recruiting CD133 positive cells was associated with increased intrahepatic precursor cells, immunofluorescence double staining for CD133 and OV6 (a marker of intrahepatic precursor cells (oval cells)) was performed. OV6 and CD133 positive cells were detected in both groups at 3 days after 85% PH. Interestingly, CD133 positive cells appeared in the area immediately around OV6 positive cells and both CD133 and OV6 positive cells were increased in animals receiving AF combination therapy (Fig. 5a). To determine whether oval cells were undergoing proliferation immunofluorescence double staining for Ki67 and OV6 was performed. Figure 5b shows that some OV6 positive cells stained with Ki67. These results suggest that recruitment of CD133 stem cells in the remnant liver was associated with intrahepatic oval cell proliferation.

### Recruitment of CD133 stem cells in the remnant liver paralleled the increased SDF-1, CXCR4 and HGF expression

**Figure 6:**
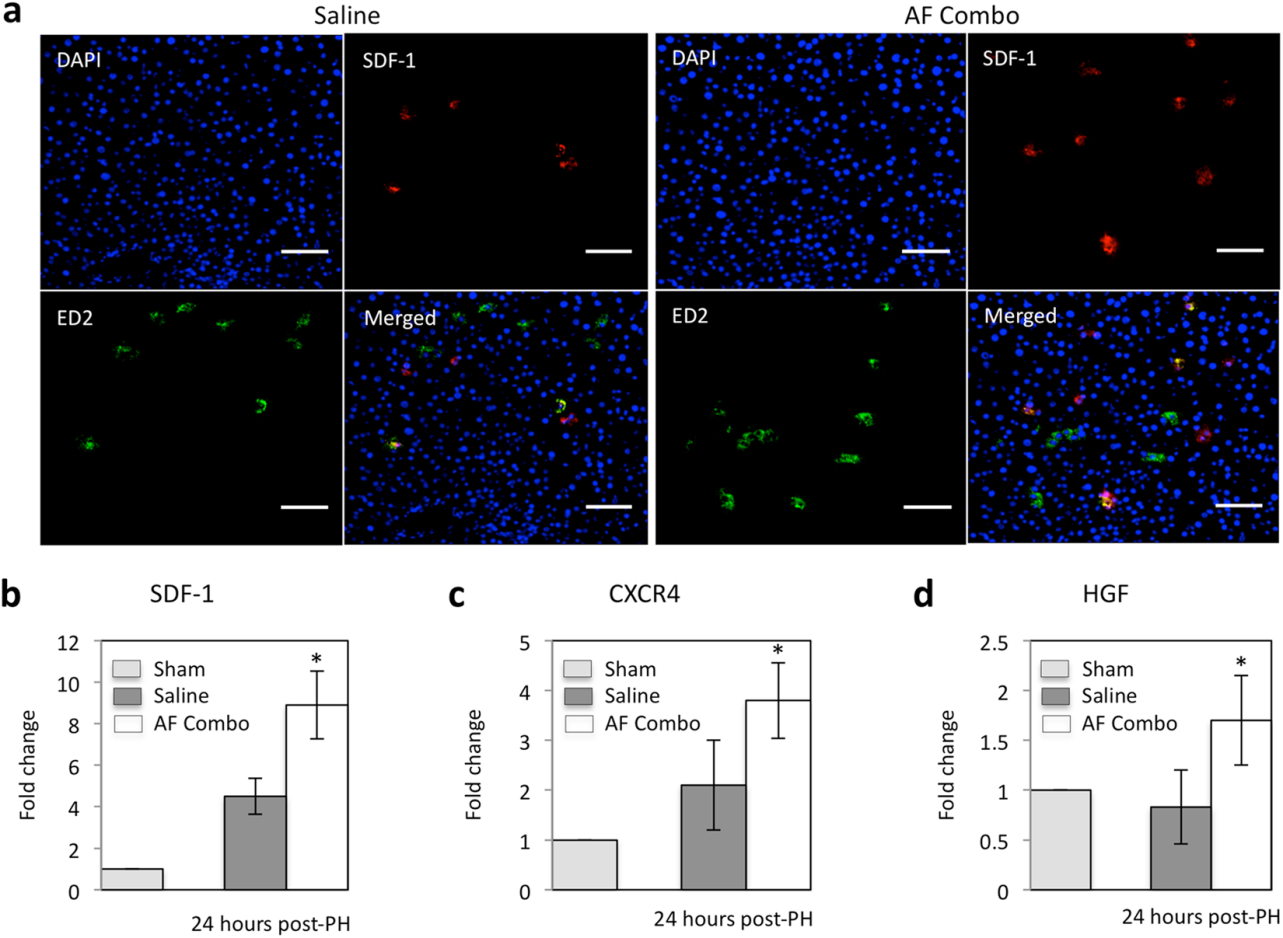
Increased SDF-1 expression paralleled the increased CXCR4 and HGF expression in the remnant liver after 85% PH. (**a**) Double staining for ED2 (CD163), a marker of Kuffer cells, and SDF-1 was performed by immunofluorescent stains using frozen sections at 3 days after 85% PH. SDF-1 was stained by anti-SDF-1 antibody and Cy3-conjugated donkey anti rabbit IgG (red), whereas ED2 was stained by FITC conjugated anti mouse IgG (green). Cell nuclei were stained blue with DAPI. The number of SDF-1 positive cells was significantly increased and about 50% SDF-1 positive cells co-stained with ED2 in animals receiving AF combination therapy. Scale bar: 100 μm. (**b**,**c** and **d**) Semi-quantitative RT-PCR analysis of SDF-1, CXCR4 and HGF expression in the remnant liver at 24 hours after 85% PH. SDF-1 and CXCR4 expression were significantly increased in animals after 85% PH, especially in animals receiving AF combination. HGF expression was only increased in animals receiving AF combination therapy. Data represent mean ± SE of n = 3 or 4 animals per group. *p < 0.05.

Stromal cell-derived factor-1 (SDF-1), a CXC chemokine family member, can mobilize endogenous stem cells into injured tissues^33,34^ through binding CXCR4 receptor. To determine whether AF combination therapy increased SDF-1 expression, immunofluorescence staining and semi-quantitative reverse transcription PCR were performed. Figure 6a shows that SDF-1 positive cells were significantly increased in tissue sections from remnant livers of AF treated animals compared with control animals at 3 days after 85% PH. Immunofluorescence double staining demonstrated about 50% of SDF-1 positive cells co-staining with ED2, a marker of resident macrophages.

SDF-1 mRNA was semi-quantitatively analyzed in remnant livers at 3 days after 85% PH by reverse transcription PCR. Figure 6b shows that SDF-1 mRNA was increased 4 fold in saline control animals and 8 fold in AF treated animals compared with animals without PH. Similarly, CXCR4 mRNA was also increased in remnant livers after 85% PH and the levels of CXCR4 mRNA were significantly higher in animals treated with AF combination (Fig. 6c). Notably, the higher levels of SDF-1 and CXCR4 mRNA in remnant livers of AF treated animals paralleled the increased HGF expression (Fig. 6d).

HGF is a cytokine that plays a crucial role in tissue regeneration, stimulating cell growth, cell motility, and morphogenesis. Bone marrow progenitor cells, which are recruited to the liver after injury and after partial hepatectomy^15^, are rich in HGF. The increased expression of HGF at both mRNA (Fig. 6d) and protein (Supplemental Fig. 1c) levels in remnant livers of AF treated animals suggests the recruitment of bone marrow progenitor cells into the remnant liver after 85% PH.

## Discussion

The current studies demonstrate the beneficial effect of AF combination therapy in liver regeneration in this rat model of extensive liver resection. All rats survived after 85% partial hepatectomy and this survival was associated with mobilization of Lin-CD34+ and Lin-CD133+ bone marrow stem cells in circulation and engraftment of CD133+ stem cells in the remnant liver, but decreased levels of serum albumin. Of greatest importance, there was significant increase in mobilization and recruitment of stem cells into the remnant liver, marked promotion of liver regeneration and improvement of serum albumin when animals were treated with AF combination.

AMD3100 (Plerixafor or Mozobil), is a direct antagonist of CXCR4 and has been used clinically to drive hematopoietic stem cells out of the BM into the peripheral blood of humans where they can be recovered and preserved until the completion of ablative irradiation and/or chemotherapy^35^. FK506, the other component of our combination therapy, is a calcineurin inhibitor and a potent immunosuppressive drug. FK506 given at low dose (0.1 mg/kg), but not standard immunosuppressive dosages increases SDF-1 expression in liver allografts^24^ and skin wound sites^27^. Our previous studies demonstrated a synergy of AMD3100 and low-dose FK506 in mobilization and recruitment of bone marrow CD34+ and CD133+ stem cells^24–27^. AMD3100 treatment increases the availability of Lin-CD34+ and Lin-CD133+ stem cells in the circulation, while low dose FK506 may enrich stem cells in allografts^24,25^ or wound sites^27^ via increased expression of SDF-1. Combined treatment with AMD3100 plus low-dose FK506 (AF combination) for only the first week resulted in long-term liver transplant acceptance^24^. This acceptance was associated with partial host replacement of donor liver tissue and down regulation of the immune response. Similarly, short-term treatment with AF combination and repeat dosing at 1, 2 and 3 months result in long-term kidney allograft survival in rats^25^ and swine^26^. Applying this combined treatment in other models, we found significantly faster skin wound healing with regeneration of hair follicles and less scar formation^27^. Lineage tracing demonstrated the critical role of bone marrow derived CD133 stem cells in enhanced capillary and hair follicle neogenesis, Thus a stem cell mobilizing strategy using AF combination to promote tissue regeneration had been developed and this was tested in the present study using a rat model of extensive liver resection.

The reduction in liver size after hepatectomy results in an immediate increase in the portal vein (PV) pressure, leading to sinusoidal shear stress, considered as an important initiating factor for regeneration. In response to shear stress, hepatocytes are primed, through the release of inflammatory cytokines (IL-6/TNF-a), to increase the expression of immediate early genes and increase the activation of transcriptional factors (STAT3/NFkB). These hepatocytes respond to growth factors, including hepatocyte growth factor, epidermal growth factor, and transforming growth factor-a. However, shear stress due to exceeding the blood flow capacity of the small liver after extensive resection can cause flow injury to sinusoidal endothelial cells, disruption of sinusoidal endothelial cells, loss of microvilli of hepatocytes and oxidative stress^36–38^. Over stimulation of TNF-α and increased sensitivity to endotoxin (lipopolysacharides) have been proposed^39^ as possible mechanisms for the intracellular production of ROS and lipid peroxidation known to follow 70% PH. The oxidative injury is more dramatic after large (87%) hepatectomy^40^. Thus, excessive oxidative stress may suppress the regenerative capacity of the liver and is one of the main causes of liver failure. Rapid regeneration of the remnant liver after extended hepatectomy is essential to prevent liver failure.

In our experiments, serum levels of AST and ALT were dramatically increased and albumin levels reduced 20% in animals after 85% partial hepatectomy indicating severe injury of the remnant liver. AF combination treatment did not eliminate liver injury. One possibility is that the AF combination treatment was given at 6 hours after partial hepatectomy and the remnant liver had already been damaged due to increased portal pressure and oxidative stress. Mobilization of endogenous bone marrow stem cells before surgery may prevent/reduce liver injury. Nevertheless, AF combination treatment promoted liver regeneration and increased serum levels of albumin, indicating the improvement of liver function.

It is well established that the mobilization and recruitment of bone marrow progenitors is necessary for normal liver regeneration. In fact, DeLeve *et al*.^17^ showed that expression of SDF-1 doubles in liver and liver sinusoidal endothelial cells (LSECs) after 70% partial hepatectomy. Upregulation of SDF-1 expression increases mobilization of CXCR7+ BM progenitors of LSECs to the circulation, and engraftment of CXCR7+ BM progenitors in the liver and promotes liver regeneration. Inhibition of SDF-1 expression in liver through VEGF knockdown impairs BM progenitors recruitment and inhibits liver regeneration after partial hepatectomy^17^. In our rat model of extensive (85%) liver resection, the SDF-1 expression was significantly increased in the remnant liver, and the increased SDF-1 expression was associated with mobilization of Lin-CD34+ and Lin-CD133+ stem cells in peripheral blood, recruitment of CD133+ stem cells in the remnant liver and subsequent liver regeneration. This finding may explain why all animals can survive after 85% partial hepatectomy. Beyond that, it is clear from our flow cytometric analysis and immunohistological studies that Lin-CD34+ and Lin-CD133+ stem cells in blood and CD133+ stem cells in the remnant liver are significantly higher in animals with AF combination therapy. Interestingly, the increased CD133+ stem cells in the remnant liver paralleled the increased SDF-1, CXCR4 and HGF expression and about 50% of SDF-1 positive cells co-stained with ED2, a marker of Kupffer cells. In liver transplantation and skin wound healing models, we have shown that low-dose FK-506 increases SDF-1 in macrophages^24,27^. As the AMD3100 effect wanes after dosing, the remnant liver SDF-1 is a strong attractant to binding by the CXCR4+ cells.

**Figure 7:**
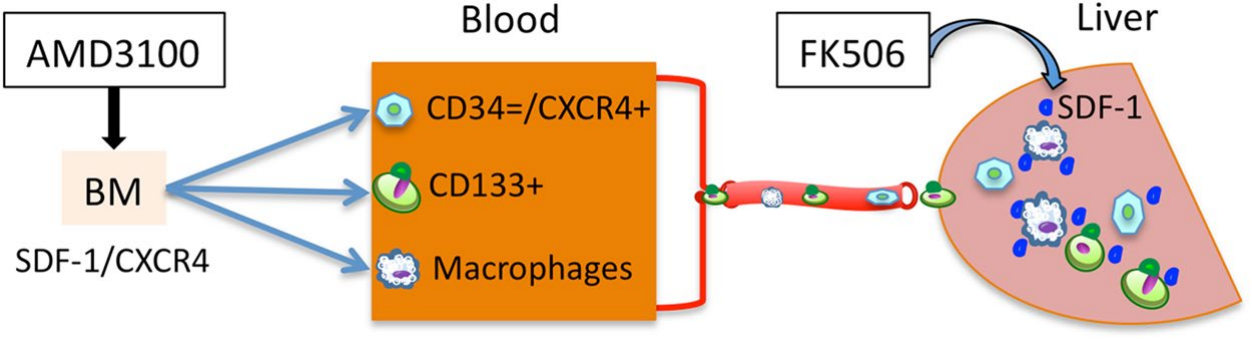
Schematic representation of therapeutic mechanism of AF combination treatment in liver regeneration. AMD3100, (**a**) CXCR4 antagonist, blocks the SDF-1/CXCR4 reaction liberating cells to enter the circulation. Low-dose FK506 alone increased SDF-1 presence in the remnant liver. Macrophages are liberated from the bone marrow by low-dose FK506 and recruited to the injured sites. These macrophages carry surface SDF-1, augmenting stem cell graft incorporation. As the AMD3100 effect wanes after dosing, the remnant liver SDF-1 is a strong attractant to binding by the CXCR4+ cells. Therefore AMD3100 treatment increases the availability of stem cells in the circulation, while low dose FK506 may enrich stem cells in the liver via increased expression of SDF-1. Recruited stem cells may promote regeneration of the remnant liver after partial hepatectomy.

Thus the mechanism of the benefit of AF combination treatment is that it not only increased the availability of stem cells in peripheral blood through liberating stem cells to move out of the bone marrow via blocking the interaction of CXCR4 and SDF-1, but also promoted the recruitment of stem cells into the remnant liver by increasing SDF-1 expression (Fig. 7). As a consequence, AF combination therapy promotes liver regeneration and improves the recovery of liver function.

In regard to mesenchymal stem cells (MSCs), no specific marker has yet been found. However, it has been demonstrated that the CD133 positive cell fraction in blood contains more MSCs with high proliferative potential and plasticity^41^. CD133 is also a marker of early endothelial progenitor cells (CD31+ CD133+ )^42^. Thus CD133+ stem cells mobilized with AF combination may contain both MSCs and endothelial progenitors. Accumulation of bone marrow-derived MSCs and endothelial progenitor cells in the remnant liver are likely to promote liver regeneration. Immunohistochemistry staining for Ki67, a cellular marker for proliferation and BrdU incorporation assays provided direct evidence that mobilizing bone marrow stem cells promote increased liver regeneration after extended partial hepatectomy.

In a previous study using a small liver transplantation model, it was found that bone marrow stem cells mobilized by AF combination treatment can differentiate into liver cells and partially repopulate liver allografts^24^. Although mobilized CD133+ stem cells may generate different components of liver tissue, we chose to direct our focus to liver regeneration and recovery of liver function. Whereas others have reported the role of bone marrow stem/progenitor cells in liver regeneration, this study expands on that to demonstrate that pharmacological mobilization of endogenous bone marrow stem cells can enhance this natural process to promote liver regeneration and improve liver function after extensive hepatectomy.

## Materials and Methods

### Animals

Male and female Lewis rats were purchased from Harlan Sprague-Dawley (Indianapolis, IN) and used at 8 to10 weeks of age with body weight between 220 to 250 grams. Animals were maintained in pathogen-free facility of Johns Hopkins University School of Medicine and all animal experiments were performed in accordance with the United States National Institutes of Health (NIH) guidance. All animal protocols were reviewed and approved by the Johns Hopkins University Animal Care and Use Committee.

### 85% Partial Hepatectomy

Left lateral lobe (LL), left and right median lobe (LML and RML), both caudate lobes (SCL and ICL) and right inferior lobe (RIL) were ligated at the pedicle bottom using 4-0 or 5-0 sutures and resected. Only the right superior lobe (RSL) was retained (Fig. 1a,b). A very small triangle-shaped liver tissue at the bottom of right median lobe near the vena cava was saved to avoid vena cava stenosis. The remnant liver was about 15% of the normal size. Animals with bleeding during surgery were excluded from studies.

### Experimental Groups

Fifty-three animals with 85% partial hepatectomy were randomly divided into two groups: 1. AF treatment group (n = 30): animals receiving AF combination therapy (AMD3100 1 mg/kg and FK506 0.1 mg/kg, subcutaneous injection) at 6, 24 and 48 hours after surgery (Fig. 1c); 2. Control group (n = 23): animals receiving the same volume of saline (2 ml/kg) at 6, 24 and 48 hours after surgery. In selected experiments, bromodeoxyuridine (5-bromo-2-deoxyuridine, BrdU, abcom) for detection of proliferating cells was administered intraperitoneally in a dose of 50 mg/kg two hours before sacrificing. At least four animals from the AF treatment group or three animals from the control group were sacrificed and liver tissues and blood were collected and stored for further analysis at 1, 2, 3, 5, 7, 10, 15 days post partial hepatectomy. In addition, a replication study to confirm our findings was performed by using an additional twenty rats and these animals were sacrificed for collecting liver tissues and blood at 3 and 15 days post partial hepatectomy (Supplemental Fig. 1).

### Analysis of serum alanine aminotransaminase and aspartate aminotransaminase activity and albumin levels

Liver injury was estimated by measurement of serum alanine aminotransaminase (ALT) and aspartate aminotransaminase (AST) levels using an automated enzyme assay. Liver function was evaluated by measurement of albumin levels in blood. These analyses were performed in the Animal Phenotyping and Pathology Core at Johns Hopkins University School of Medicine.

### Preparation of peripheral blood mononuclear cells (PBMC)

Mononuclear cells were isolated from peripheral blood at 24, 48 and 72 hours after partial hepatectomy by Ficoll-Hypaque (1.077 g/L, Sigma-Aldrich, St. Louis, MO) density gradient centrifugation. Peripheral blood mononuclear cells (PBMCs) were harvested from the interface, then washed once with phosphate-buffered saline and once with RPMI 1640 (Invitrogen, Carlsbad, CA) and centrifuged at 400 g for 5 min.

### Flow cytometry

Single-cell suspensions (1 × 10^6^) of PBMC were analyzed for expression of lineage-negative (Lin−) CD34+ and CD133+ stem cell markers as we described^24,25^. All antibodies used were from commercial sources: CD133 (Abcam, Cambridge, U.K.); CD34 (R&D Systems, Minneapolis, MN); Cy3 (rabbit), FITC (goat) (eBioscience). Nonspecific antibody binding was blocked with donkey and mouse serum (Sigma-Aldrich) for 30 min. Cells were incubated with antibodies for 1 h at 4 °C, and the positive cells were counted by flow cytometry (fluorescence-activated cell sorting [FACS]) using CELLQuest software (Becton Dickinson, Franklin Lakes, NJ).

### Immunohistochemistry

Cut sections of 5 µm were prepared from formalin-fixed paraffin-embedded tissue for H&E staining or frozen tissue for immunohistochemistry staining. Frozen sections were fixed with acetone at −20 °C for 10 min and dried for 1 h at room temperature. BrdU staining was performed by using a BrdU *in situ* detection kit (BD Biosciences, San Jose, CA). The streptavidin–biotin–peroxidase method with the DAKO Kit (DAKO, Carpinteria, CA) was used to detect Ki67 and CD133 antigens. After inactivation of endogenous peroxidase and blocking of nonspecific antibody binding, the specimens were treated with anti-Ki67 (1:500, ab16667, Cambridge, MA) or biotinylated antibodies specific for CD133 (1:100, ab19898; Abcam) at 4 °C overnight. For Ki-67 staining, the tissue sections were subsequently incubated with biotin-conjugated goat anti rabbit IgG (1:200, #14708 S Cell Signalling, Danvers, MA) for 30 minutes at room temperature. Diaminobenzidine tetrahydrochloride (5 min, D4293, Sigma-Aldrich, St. Louis, MO) was used as the chromogen, and Mayer’s Hematoxylin (30 s, Dako, S3309) was used for counterstaining.

### Immunofluorescence staining

Frozen sections (5-μm) were used for immunofluorenscence double staining. A Tris-based buffer containing 0.5% casein, 5% normal rat and rabbit serum was used for blocking non-specific background and dilution of antibodies. Sections were incubated for 45 minutes at room temperature with a mixture of a mouse antibody to rat/human OV6 (1:200, MAB2020, R&D, Minneapolis, MN) and a rabbit antibody to CD133 (1:100, ab19898; Abcam) or a rabbit antibody to Ki67 (1:500), followed by FITC-donkey anti mouse IgG (1:200, Jaskson ImmunoResearch Lab) or Cy3-donkey anti rabbit IgG (1:200, Jaskson ImmunoResearch Lab) for 1 hour at room temperature. For SDF-1 and ED2 staining, sections were incubated for 45 minutes at room temperature with mixture of a FITC labeled mouse antibody to rat CD163 (ED2) (1:200, MCA342F, Bio-Rad,Hercules, CA) and a rabbit antibody to SDF-1 followed by Cy3-donkey anti- rabbit IgG (1:200) for 1 hour at room temperature. Cell nuclei were stained blue with DAPI. Tissue sections were analyzed by confocal fluorescence microscopy.

### Semi-quantitative reverse transcription (RT)-PCR analysis

Liver specimens were kept frozen at −80 °C until homogenized for RNA extraction using the TRIzol Reagent (Invitrogen, Carlsbad, CA). First-strand cDNA synthesis was then performed on 5 μg of total RNA using the Superscript First-Strand Synthesis system for RT-PCR (Invitrogen, Carlsbad, CA) according to the manufacturer’s instructions. RT-PCR for mRNA expression of SDF-1, CXCR4 and HGF were performed according to the methods we described previously^24^.

### Statistics

Data were presented as the mean ± SD. Dichotomous variables were presented as both number and percentage values. Flow cytometry data were analyzed using the Student’s t test (two-tailed), with dichotomous variables analyzed by the Fisher’s exact test (two-tailed). All analyses were performed using SPSS® (SPSS; Chicago, IL). p < 0.05 was considered significant.

## Electronic supplementary material

Supplemental Figure 1
