# Supplementary material for: Pharmacological Mobilization of Endogenous Bone Marrow Stem Cells Promotes Liver Regeneration after Extensive Liver Resection in Rats
Source: Sci Rep. 2018 Feb 26;8:3587. doi: 10.1038/s41598-018-21961-2 (PMC5827664; doi:10.1038/s41598-018-21961-2)
Supplement: Supplementary file 1 — Supplemental Figure 1 [file 41598_2018_21961_MOESM1_ESM.pdf]

Pharmacological Mobilization of Endogenous Bone Marrow Stem Cells Promotes Liver Regeneration after Extensive Liver Resection in Rats

Rujun Zhai[1, 2], Yongchun Wang[2], Le Qi[2], George Melville Williams[2], Bin Gao[3], Guang Song[4], James F. Burdick[2], Zhaoli Sun[2*]

Supplemental Figure 1

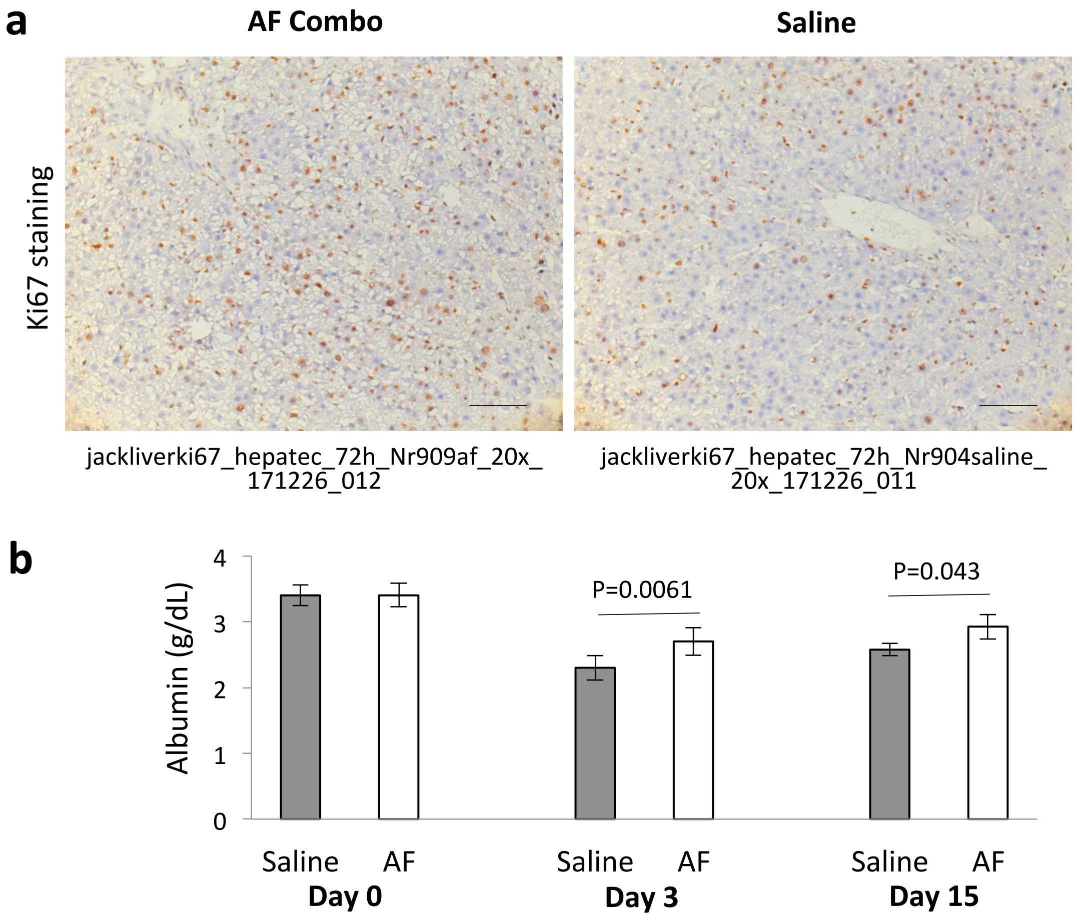

c

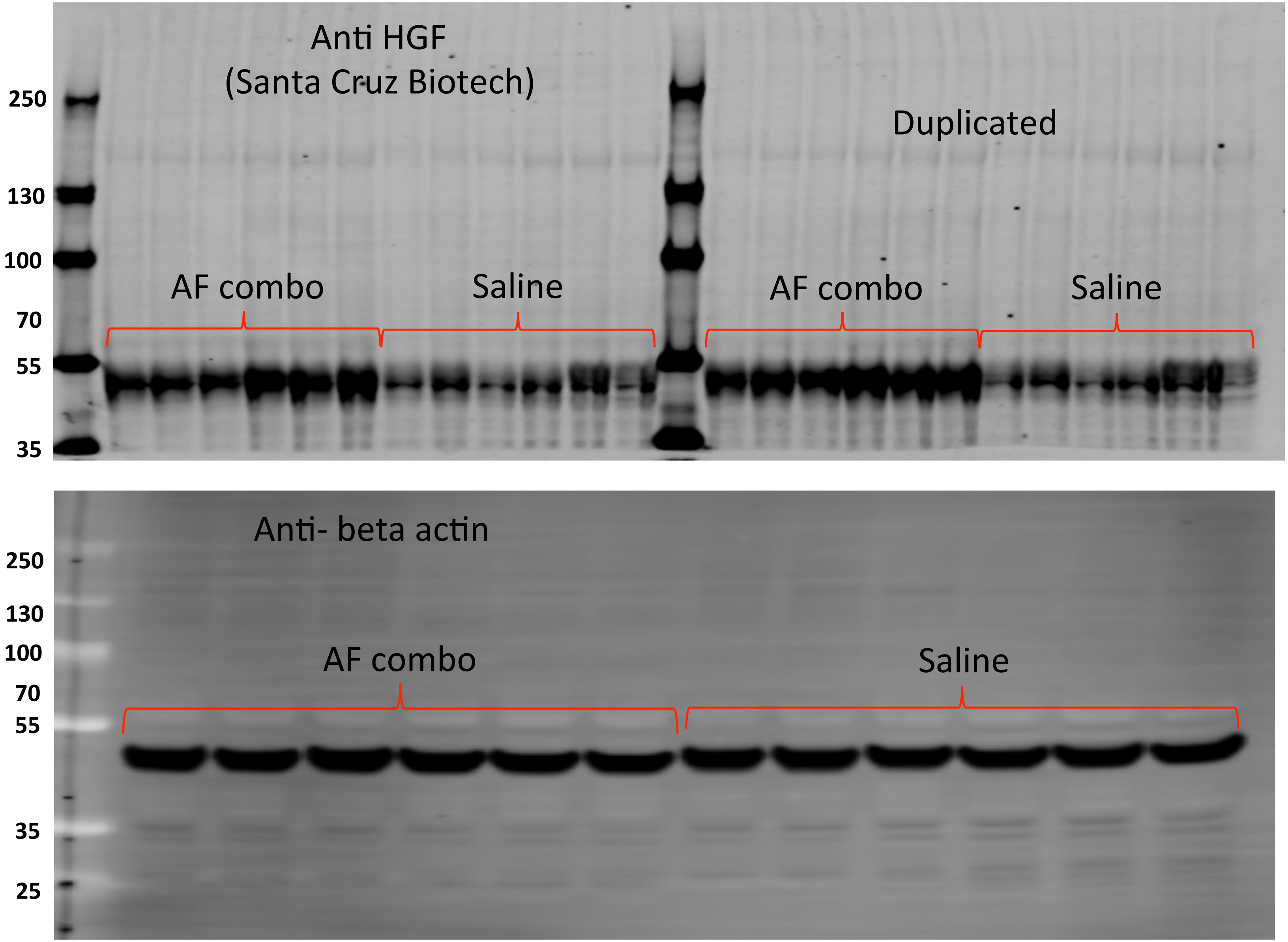
Anti HGF
(Santa Cruz Biotech)
Duplicated
250
130
100
70
55
35
AF combo
Saline
AF combo
Saline
Anti- beta actin
250
130
100
70
55
35
25
AF combo
Saline

Figure S1. A replication study. Twenty animals with 85% partial hepatectomy were randomly divided into two groups: 1. AF treatment group (n=10): animals receiving AF combination therapy (AMD3100 1mg/kg and FK506 0.1mg/kg, subcutaneous injection) at 6, 24 and 48 hours after surgery; 2. Control group (n=10): animals receiving the same volume of saline (2ml/kg) at 6, 24 and 48 hours after surgery. All animals from each group were sacrificed and liver tissues and blood were collected and stored for further analysis at 3 days and 15 days post partial hepatectomy.(**a**) The number of Ki67 positive cells was significantly higher in tissue sections from animals treated with the AF combination at 72 hours after 85% PH. Scale bar=100µm. (**b**) Animals treated with the AF combination exhibited significantly higher levels of albumin at day 3 (n=6 per group) which remained higher at day 15 (n=4 per group) after 85% PH. (**c**) HGF expression was significantly increased in remnant livers of AF treated animals at day 3 after 85% PH .
